# Supplementary material for: Targeted Gene Delivery into the Mammalian Inner Ear Using Synthetic Serotypes of Adeno-Associated Virus Vectors
Source: Mol Ther Methods Clin Dev. 2019 Jan 11;13:197–204. doi: 10.1016/j.omtm.2019.01.002 (PMC6374519; doi:10.1016/j.omtm.2019.01.002)
Supplement: Document S1. Figures S1–S3 [file mmc1.pdf]

OMTM, Volume *13*

Supplemental Information

Targeted Gene Delivery into the Mammalian Inner Ear Using Synthetic Serotypes of Adeno-Associated Virus Vectors

Min-A Kim, Nari Ryu, Hye-Min Kim, Ye-Ri Kim, Byeonghyeon Lee, Tae-Jun Kwon, Jinwoong Bok, and Un-Kyung Kim

Supplementary Figures

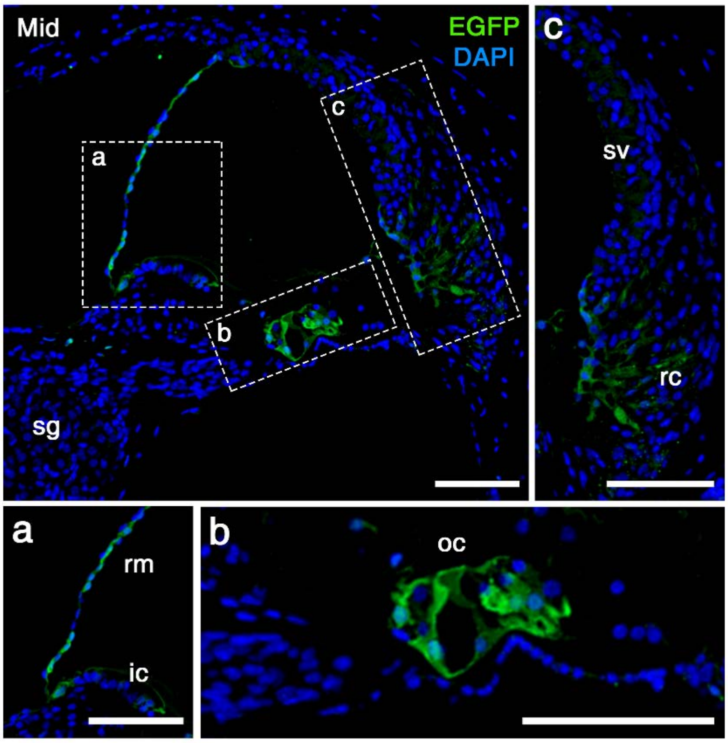

Supplementary Figure 1. Transduction of rAAV2/DJ into murine cochlea. EGFP immunoreactivity (green) in cross-sections of cochlear middle turns in 3-week-old mice infected with rAAV2/DJ. High-magnification images of Reissner's membrane, interdental cells, organ of Corti, root cells, and stria vascularis are shown (a–c, respectively). Cell nuclei were stained with DAPI (blue). ic, interdental cell; oc, organ of Corti; rc, root cell; rm, Reissner's membrane; sg, spiral ganglion; sv, stria vascularis. Scale bars represent 100 μm.

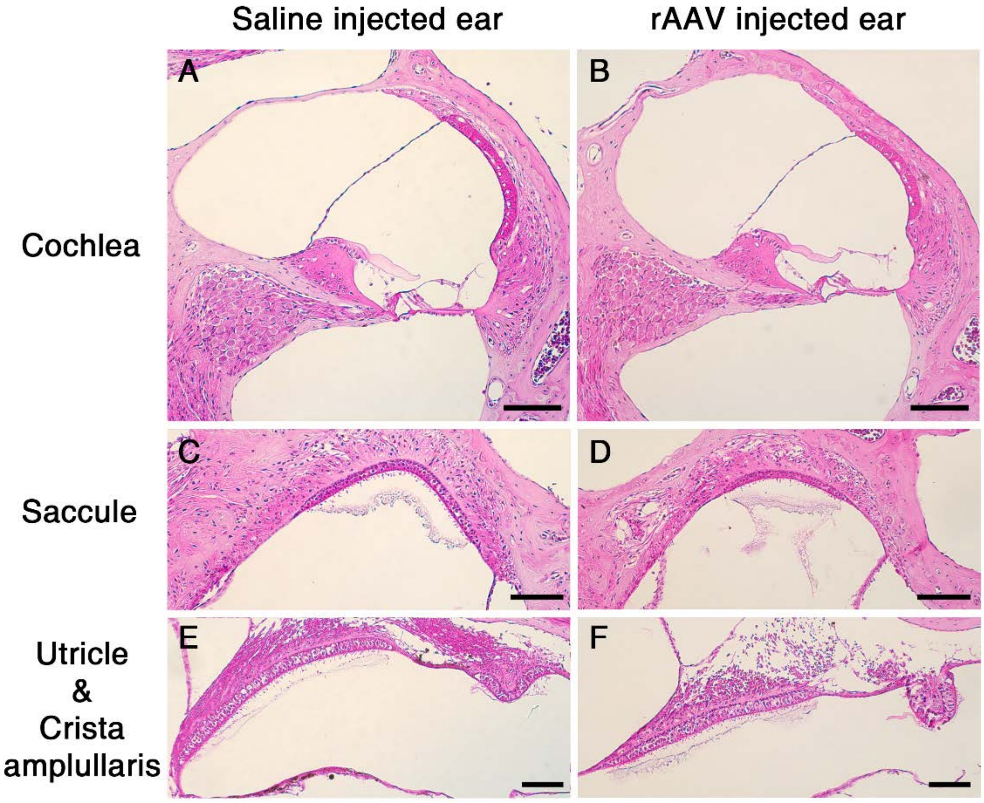

Supplementary Figure 2. Histological observations of the utricle, saccule, and crista ampullaris. Cross-sections of the (A and B) cochlear middle turn, (C and D) saccule, (E and F) utricle and crista ampullaris from saline and rAAV injected ears of mice were obtained at 3 weeks of age and stained with hematoxylin-eosin. Representative images of three replicates each are shown. Scale bars: 100 µm.

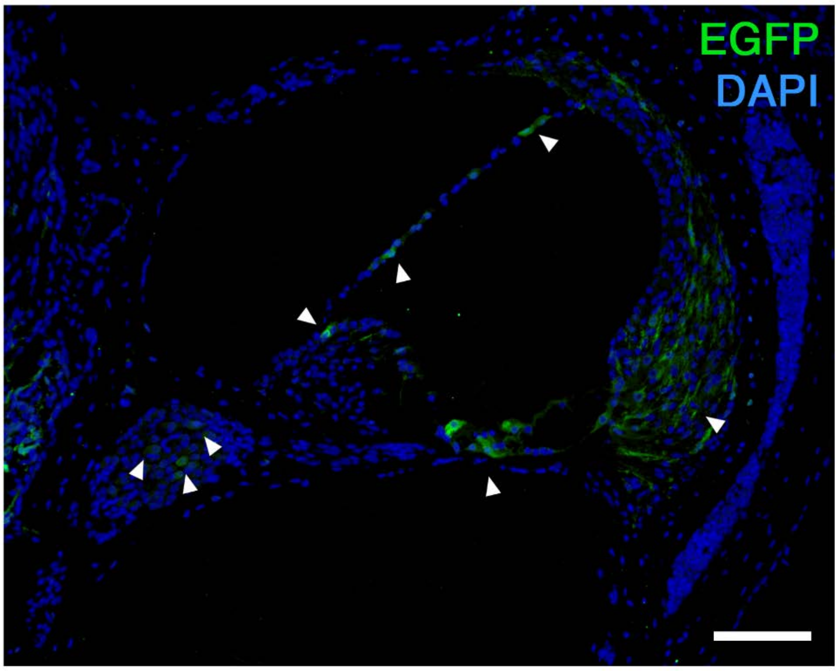

Supplementary Figure 3. EGFP expression in the cochlea infected by rAAV2/PHP.B. EGFP immunoreactivity (green) in cross-sections of cochlear basal turns in mice 3 weeks of age are shown. Nuclei were stained with DAPI (blue). White arrowheads indicate representative EGFP-expressing cells. Scale bar represents 100 µm.
