# Supplementary material for: Targeted Gene Delivery into the Mammalian Inner Ear Using Synthetic Serotypes of Adeno-Associated Virus Vectors
Source: Mol Ther Methods Clin Dev. 2019 Jan 11;13:197–204. doi: 10.1016/j.omtm.2019.01.002 (PMC6374519; doi:10.1016/j.omtm.2019.01.002)
Supplement: Document S2. Article plus Supplemental Information [file mmc2.pdf]

Molecular Therapy
Methods & Clinical Development
Original Article

Targeted Gene Delivery into the Mammalian Inner Ear Using Synthetic Serotypes of Adeno-Associated Virus Vectors

Min-A Kim,[1,2,7] Nari Ryu,[1,2,7] Hye-Min Kim,[1,2] Ye-Ri Kim,[1,2] Byeonghyeon Lee,[1,2] Tae-Jun Kwon,[3] Jinwoong Bok,[4,5,6] and Un-Kyung Kim[1,2]

[1]Department of Biology, College of Natural Sciences, Kyungpook National University, Daegu 41566, Republic of Korea; [2]School of Life Sciences, BK21 Plus KNU Creative BioResearch Group, Kyungpook National University, Daegu 41566, Republic of Korea; [3]Laboratory Animal Center, Daegu-Gyeongbuk Medical Innovation Foundation (DGMIF), Daegu 41061, Republic of Korea; [4]Department of Anatomy, Yonsei University College of Medicine, Seoul 03722, Republic of Korea; [5]Department of Otorhinolaryngology, Yonsei University College of Medicine, Seoul 03722, Republic of Korea; [6]BK21 PLUS Project for Medical Science, Yonsei University College of Medicine, Seoul 03722, Republic of Korea

Targeting specific cell types in the mammalian inner ear is important for treating genetic hearing loss due to the different cell type-specific functions. Adeno-associated virus (AAV) is an efficient *in vivo* gene transfer vector, and it has demonstrated promise for treating genetic hearing loss. Although more than 100 AAV serotypes have been identified, few studies have investigated whether AAV can be distributed to specific inner ear cell types. Here we screened three EGFP-AAV reporter constructs (serotypes DJ, DJ8, and PHP.B) in the neonatal mammalian inner ear by injection via the round window membrane to determine the cellular specificity of the AAV vectors. Sensory hair cells, supporting cells, cells in Reissner's membrane, interdental cells, and root cells were successfully transduced. Hair cells in the cochlear sensory epithelial region were the most frequently transduced cell type by all tested AAV serotypes. The recombinant DJ serotype most effectively transduced a range of cell types at a high rate. Our findings provide a basis for improving treatment of hereditary hearing loss using targeted AAV-mediated gene therapy.

INTRODUCTION

Sensorineural hearing loss (SNHL) involves damage to the cochlea and the auditory nerve, and it accounts for approximately 90% of hearing loss cases, over half of which have an underlying genetic etiology.[1] The mammalian inner ear is composed of various cell types with distinct roles,[2] and mutations therein affect multiple vital cochlear functions, such as the development of the sensory organ, sound transduction in hair cells, maintenance of the high concentration of extracellular potassium and endocochlear potential, and synaptic neurotransmission between hair cells and spiral ganglion neurons.[2,3] Genetic hearing loss displays variable expressivity depending on which gene is involved and the type of mutation that is present. Several successful studies of gene therapy in SNHL mouse models have demonstrated that cochlear gene therapy is a potentially effective strategy for ameliorating hereditary hearing loss in humans.[4–9] However, despite the initial success, a system in which the therapeutic gene would be transported to the correct native locus is still to be developed.[3]

Adeno-associated virus (AAV) is an effective nonpathogenic gene transfer vector that can be used for treating hereditary hearing loss.[10] The function of an AAV vector is determined mainly by the capsid protein,[11–13] and because viral replication and packaging proteins are generally interchangeable, researchers can quickly investigate various serotypes in animal models to determine which serotype best suits their needs. For many years, only five primate AAV serotypes were available,[14–17] although Gao et al.[18,19] subsequently identified over 100 new capsid variants by searching human and non-human primate tissues. Numerous AAV serotypes have been engineered by directed evolution,[20–22] including the serotypes DJ and DJ8, which carry a heparin-binding domain and exhibit tropism for the liver and brain, similar to that of serotypes AAV8 and AAV9.[22] The engineered AAV9 variant PHP.B contains a randomized insert of seven amino acids, and it has shown broader transduction distribution following intravenous infusion into the central nervous system (CNS) of mice compared to AAV9.[23]

Although many AAV serotypes have been engineered to improve their transduction to target tissues, some do not exhibit the expected tropism patterns. Moreover, not all AAV serotypes and their respective mammalian cell surface receptors have been well

Received 19 September 2018; accepted 7 January 2019;
https://doi.org/10.1016/j.omtm.2019.01.002.

[7]These authors contributed equally to this work.

Correspondence: Un-Kyung Kim, PhD, Department of Biology, College of Natural Sciences, Kyungpook National University, Daegu 41566, Republic of Korea.
E-mail: kimuk@knu.ac.kr
Correspondence: Jinwoong Bok, PhD, Department of Anatomy, Yonsei University College of Medicine, Seoul 03722, Republic of Korea.
E-mail: bokj@yuhs.ac

This is an open access article under the CC BY license (http://creativecommons.org/licenses/by/4.0/).

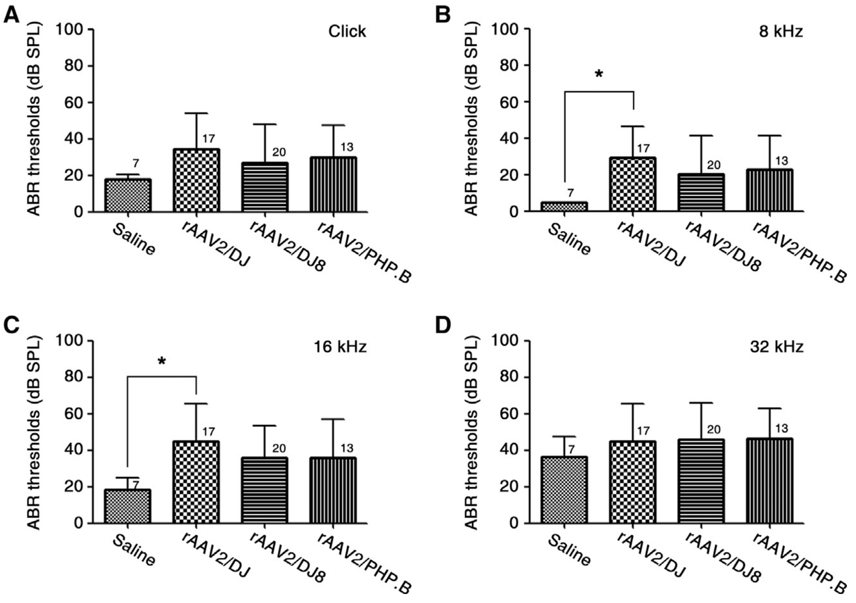

Figure 1. Comparison of Hearing Function in Mice following rAAV2/DJ-, rAAV2/DJ8-, or rAAV2/PHP.B-Mediated Gene Transfer via RWM Injection

Comparison of the ABR thresholds between rAAV-injected and saline-injected ears 3 weeks post-injection. Thresholds in response to click sound stimuli (A) and 8 kHz (B), 16 kHz (C), and 32 kHz (D) tone burst stimuli in mice injected with rAAV2/DJ, rAAV2/DJ8, or rAAV2/PHP.B are shown. Data are presented as the mean values with SD (one-way ANOVA with Bonferroni correction, *$p < 0.05$). Numbers next to symbols represent the number of biological replicates.

characterized. Thus, the successful application of newly identified serotypes to the mammalian inner ear for the treatment of hereditary hearing loss warrants further investigation. In this study, we evaluated the previously unexplored transduction distribution and efficiency of EGFP-expressing AAV2-derived vectors DJ, DJ8, and PHP.B introduced locally into the murine inner ear, aiming to provide a basis for future improvement of hereditary hearing loss treatment using targeted AAV-mediated gene therapy.

RESULTS

AAV Injection into the Inner Ears of Neonatal Mice Does Not Impair Hearing

To determine the safety of serotypes DJ, DJ8, and PHP.B for future inner ear studies and potential therapies, we first investigated hearing function by measuring auditory brainstem response (ABR) thresholds of mice at 3 weeks of age neonatally infected with AAV (Figure 1). At all frequencies measured, namely click sound stimuli and 8-, 16-, and 32-kHz tone burst stimuli, the ABR thresholds did not differ significantly among the inner ears of mice injected with recombinant AAV (rAAV)2/DJ, rAAV2/DJ8, or rAAV2/PHP.B. There was only a slight difference between the inner ears of the mice injected with saline and those of the mice injected with rAAV2/DJ at 8 and 16 kHz (*$p < 0.05$; Figures 1B and 1C). Despite the variability in mice injected with the rAAV vectors, we observed that mice generally show normal hearing with strong GFP expression. Really strong GFP expression was only detected once in mice with affected hearing function compared with the mice showing normal hearing. In addition, no animals showed symptoms of vestibular disturbance, such as circling, head tilting, and abnormal gait (data not shown). Therefore, we concluded that there was no obvious toxicity affecting hearing function due to any of the vectors tested.

Transduction of Mouse Cochlear Cells with the Serotypes DJ, DJ8, and PHP.B

To define the extent of tissue tropism of serotypes DJ, DJ8, and PHP.B, we analyzed EGFP expression by immunostaining the inner ears of 3-week-old mice infected with rAAV vectors. Modiolar cross-sections of the cochleae showed wide distribution of EGFP expression in diverse inner ear cell types and locations, including sensory epithelial and supporting cells, stria vascularis, spiral ganglion, spiral ligaments, tectorial membrane, Reissner's membrane, and spiral limbus (Figure 2).

The rAAV2/DJ infected non-sensory cells, including the supporting cells, Reissner's membrane, interdental cells, and root cells (Figure S1). Distribution of EGFP was more prominent in the organ of Corti and Reissner's membrane than in interdental and root cells. In contrast, no EGFP expression was observed in the spiral ganglion, stria vascularis, or spiral ligaments. Such a pattern of EGFP expression was also identified throughout all cochlear turns from apex to base, with no differences in transduction efficiency across tonotopic positions (Figures 2A, 2E, and 2I). rAAV2/DJ8 successfully transduced multiple inner ear cell types, with more prominent expression in the inner and outer hair cells and weaker expression in Reissner's membrane and root cells (Figures 2B, 2F, and 2J); in many sections, EGFP-positive cells were not detected in Reissner's membrane. We also observed much weaker EGFP expression in root cells in the apical than in the basal turns. Serotype rAAV2/PHP.B successfully transduced cochlear hair cells and infected cells of Reissner's membrane and the spiral ganglion less effectively (Figures 2C, 2G, and 2K). In addition, no EGFP expression was observed in the root cells after rAAV2/PHP.B transduction. Overall, rAAV2/DJ, DJ8, and PHP.B successfully transduced sensory epithelial cells (inner and outer hair cells), as shown by relatively strong EGFP expression (Figure 3). Cochlear and vestibular structures were intact and all major cell types present, suggesting that rAAV transduction did not cause serious toxicity or inner ear malformation (Figure S2). In addition, we observed no EGFP immunoreactivity in the saline-injected inner ears (Figures 2D, 2H, and 2L).

All tested rAAV serotypes transduced the sensory cells as shown by relatively strong EGFP expression. The extent of hair cell rAAV

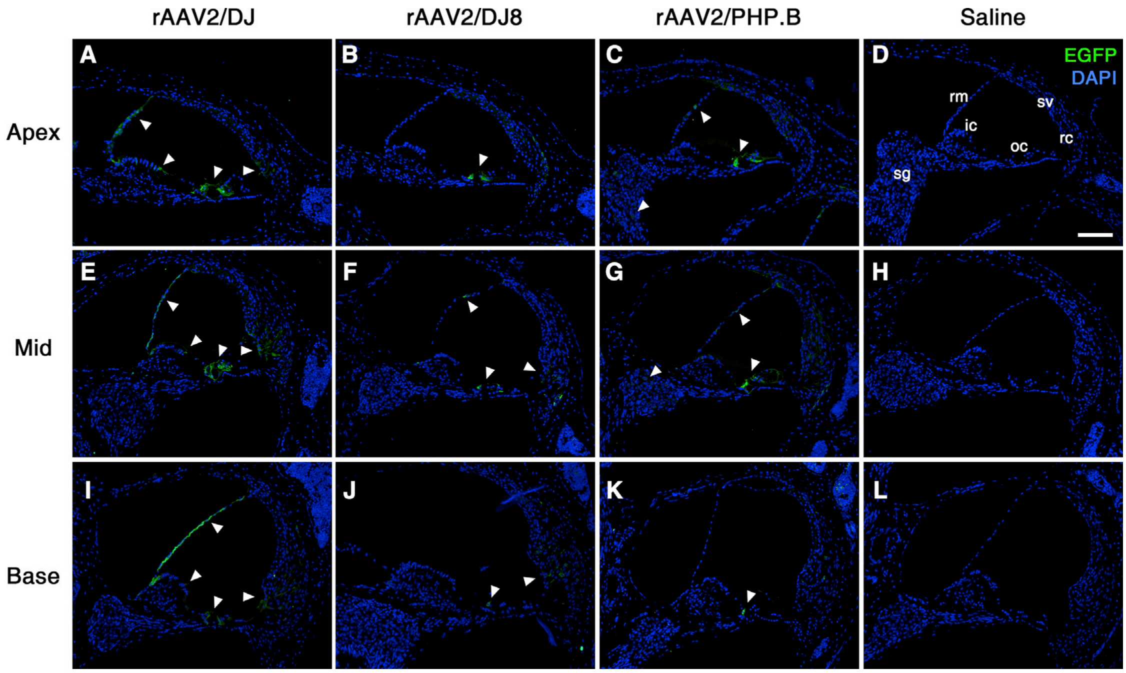

Figure 2. Transduction of rAAV Serotypes into Murine Cochlea

EGFP immunoreactivity (green) in cross-sections of three different cochlear turns: apex (A–D), mid (E–H), and base (I–L) in infected 3-week-old mice. Cell nuclei were stained with DAPI (blue). White arrowheads indicate representative EGFP-expressing cells. Representative images of six, four, three, and three replicates, respectively, are shown. ic, interdental cell; oc, organ of Corti; rc, root cell; rm, Reissner's membrane; sg, spiral ganglion; sv, stria vascularis. Scale bar represents 100 μm.

transduction was next investigated by quantitating EGFP-positive cells in cochlear whole-mount preparations 3 weeks post-infection (Figure 4). With inoculation of rAAV2/DJ, the average percentages of EGFP-positive inner hair cells in the apical, middle, and basal turns were 52%, 41%, and 57%, respectively (Figures 4A and 4B). EGFP was widely distributed among the outer hair cells, but it showed different transduction efficiency across tonotopic positions: apex, 90%; mid, 88%; and base, 37%. The average percentages of rAAV2/DJ8-positive inner hair cells were 59%, 24%, and 10% in the apical, middle, and basal cochlear turns, respectively (Figures 4A and 4C). Expression of EGFP in the outer hair cells was very limited after rAAV/DJ8 transduction. Infection with rAAV2/PHP.B also led to variable efficiency of the transduction of hair cells across tonotopic positions: apex, 86%; mid, 81%; and base, 62% in the inner hair cells; and apex, 63%; mid, 31%; and base, 16% in the outer hair cells (Figures 4A and 4D). In summary, rAAV2/DJ exhibited highly efficient transduction of outer hair cells, whereas serotypes DJ8 and PHP.B showed preferential infection of inner hair cells, with the most prominent expression in the apical region.

rAAV Transduction in Mouse Vestibules

The vestibule is a significant target for inner ear gene transfer due to the prevalence of vestibular dysfunction increasing globally, with no biological treatments currently being available.[24] Therefore, we determined the efficiency of rAAV transduction in the vestibular organ (Figure 5). We observed notable rAAV2/DJ transduction in the saccule and utricle, with more prominent EGFP expression in the vestibular hair cells of the utricle (Figures 5A and 5E). In contrast, we saw limited transduction of hair cells in the crista ampullaris (Figure 5I). Mice injected with rAAV2/DJ8 yielded similar transduction profiles, although the pattern of EGFP transduction differed from that after rAAV2/DJ transduction of the cochlea (Figures 5B, 5F, and 5J). EGFP-positive cells were observed throughout the vestibular sensory epithelia in mice injected through the round window membrane (RWM) into the scala tympani with rAAV2/PHP.B, with more prominent EGFP expression in the vestibular hair cells of the saccule and utricle (Figures 5C, 5G, and 5K). In addition, EGFP expression in the epithelium of the membranous labyrinth was observed (data not shown). Moreover, we observed no EGFP immunoreactivity in the saline-injected inner ears (Figures 5D, 5H, and 5L).

DISCUSSION

Recently, AAV has become a more recognized vehicle for successful gene delivery into the inner ear, facilitating gene function research and the development of treatments for hereditary hearing loss. Previous studies have validated several AAV vectors in the murine inner ear, namely AAV1, AAV2, AAV5, AAV6, AAV8, AAV9, and Anc80L65,[7,25–28] leading to substantial advancement of cochlear gene therapy investigations using mutant mouse models.[4–6,8,9,29–32] Although diverse serotypes have been evaluated in mice, the AAV1 vector has gained particular popularity in cochlear gene therapy targeting loci involved in sound transduction and development or

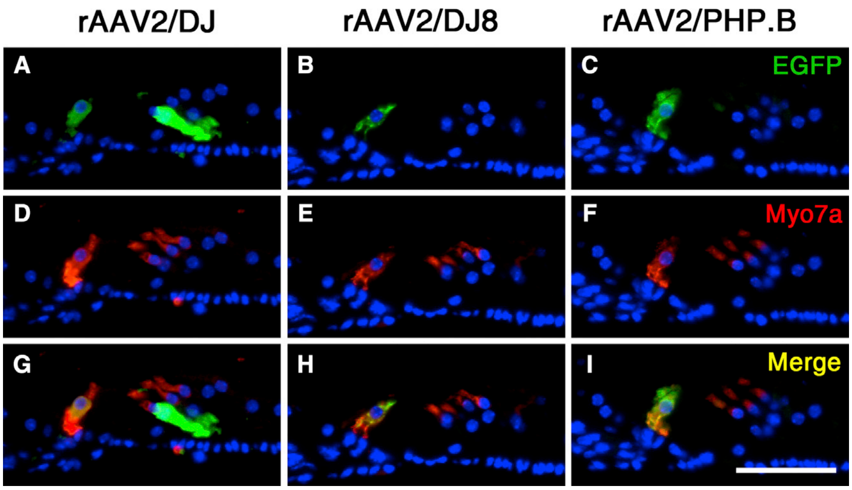

Figure 3. Transduction of rAAV Serotypes into Organ of Corti

The expressions of EGFP (green) and Myo7a (red) were assessed in cochlear cross-sections of 3-week-old mice infected with the indicated vectors: rAAV2/DJ (A, D, and G), rAAV2/DJ8 (B, E, and H), and rAAV2/PHP.B (C, F, and I). Cell nuclei were stained with DAPI (blue). Scale bar represents 50 μm.

maintenance of the stereociliary bundles.[4–6,9,29,30] Successful hearing restoration resulted from higher transduction affinities of AAV vectors for the sensory epithelial cell region, including the inner and outer hair cells and supporting cells. However, the AAV serotypes that can efficiently target specific tissues in the inner ear have not been fully studied.

In this study, we explored the tissue tropism of recombinant AAV2-derived serotypes DJ, DJ8, and PHP.B, which were generated by directed evolution and have not yet been studied for application in the inner ear. All tested AAV vectors yielded cytomegalovirus (CMV) promoter-driven EGFP expression in the inner ears of mice via a perilymphatic approach (Table 1). Overall, rAAV2/DJ, rAAV2/DJ8, and rAAV2/PHP.B transductions were relatively successful in the inner and outer hair cells, the most important cell types in the cochlea. Specifically, rAAV2/DJ robustly infected non-sensory cell types in the inner ear, including cells of Reissner's membrane, interdental cells, and root cells, whereas rAAV2/DJ8 and /PHP.B did not effectively transduce these cell types. Therefore, rAAV2/DJ may be the optimal choice for delivering functional therapeutic genes to target cells such as Reissner's membrane, interdental cells, and root cells, which are essential for diffusion barrier function by separating the cochlear duct,[33] synthesis of the tectorial membrane,[34] and ion homeostasis of the endolymph,[35] respectively. In fact, recovering specific native phenotypes using viral vectors is difficult, because many genes are expressed in the same cell type or one gene is expressed in a wide range of inner ear cell types.[36] Therefore, using rAAV vectors with tissue tropism patterns distinct from rAAV2/DJ may enable more effective and accurate gene transport to the native location.

Interestingly, the tissue tropism of rAAV2/DJ8, mediated by a mutated heparin-binding domain of rAAV2/DJ, showed a transduction pattern different from that of rAAV2/DJ in the cochlea but similar in the vestibule. The heparin-binding domain of serotype DJ, a hybrid capsid from 8 AAV serotypes (AAV2, 4, 5, 8, 9, avian, bovine, and goat), may originate from serotype 2, which possessed the heparin-binding domain.[37] Although there were several studies showing the ability of AAV serotype 2 to mediate efficient gene transfer into inner ear cell types,[5,25,38] whether heparin receptors are associated with different inner ear cell types has not been studied. Nevertheless, in this study, we observed that AAV2/DJ8 exhibited decreased transduction of Reissner's membrane cells, interdental cells, and root cells in the murine inner ear. Therefore, we expected these cell types may be involved with the heparin-binding domain. Further studies characterizing cell surface receptors of the specific inner ear cell types are needed for recovering the specific native regions of the target-deficient genes.

We observed strong EGFP expression in the spiral ligament when rAAV2/PHP.B was directly injected into the scala tympani via the RWM of neonatal mice (Figure S3). Unfortunately, the cellular distribution of EGFP in the spiral ligament was only detected once in our study, suggesting that this serotype exhibits extremely variable and limited transduction capability. With inoculation of rAAV2/DJ, we observed consistent and substantial cochlear gene transduction, whereas the efficacy of rAAV2/PHP.B varied more than that of other serotypes. Hordeaux et al.[39] reported that AAV-PHP.B, with remarkable transduction of the CNS in C57BL/6J mice by systemically administered AAV-PHP.B, did not translate to the BALB/cJ mouse strain. However, György et al.[40] have recently reported that AAV-PHP.B can efficiently transduce the inner and outer hair cells in the cochleae of neonatal mice, neonatal rats, and a juvenile non-human primate. Specifically, the transduction efficiency of hair cells was observed in CD1 mice as well as in C57BL/6 mice, indicating there was independency between mouse strain in the case of inner ear cell types, unlike in the CNS.

The tissue tropism patterns of these AAV serotypes are universally difficult to interpret because of variations in vector titers, doses, promoters, transgenes, delivery routes, and time points of administration. For example, Kim et al.[9] recently demonstrated efficient transduction of both inner and outer hair cells after the injection of rAAV2/1-CMV-GFP (1.3×10^{13} genome copies [GCs]/mL) at embryonic day 12.5, whereas Landegger et al.[38] reported strong transduction of inner hair cells but weak transduction of the outer hair cells after injection of the same construct (3.5×10^{13} GC/mL) at postnatal day 1. Despite these disparities, the effective delivery of therapeutic genes to their specific locations in the inner ear must be achieved,

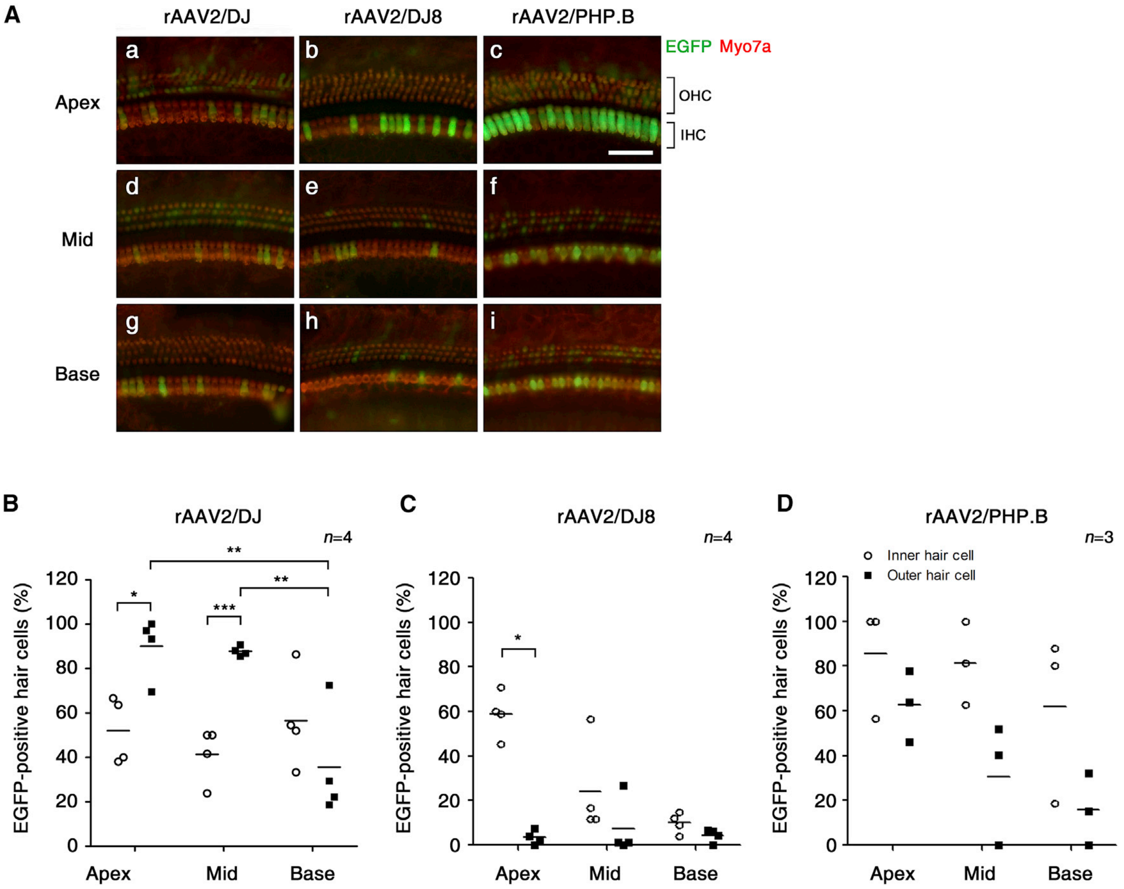

Figure 4. Distribution of rAAV2/DJ, rAAV2/DJ8, and rAAV2/PHP.B Vectors in the Sensory Epithelium of the Cochlea

Representative images of whole mounts of the organ of Corti show EGFP distribution following the delivery of rAAV vectors to mice at 3 weeks of age. (A) Tissues were stained with Myo7a (red) for labeling cochlear hair cells and imaged for native EGFP (green). IHC, inner hair cell; OHC, outer hair cell. Scale bar represents 50 μm. Quantitative comparison of hair cell transduction efficiency assessed in 200-μm segments across three different regions of the cochlea in mice at 3 weeks of age after injection with (B) rAAV2/DJ, (C) rAAV2/DJ8, or (D) rAAV2/PHP.B. Data are presented as scatter dot plots with mean values (bars) (Student's t test and one-way ANOVA with Bonferroni correction; ***$p < 0.001$, **$p < 0.01$, *$p < 0.05$).

as hearing loss is caused by numerous genetic mutations in diverse cell types of the inner ear. Successful delivery of therapeutic genes into non-sensory cells of the inner ear is as critical as their delivery to sensory hair cells, and it will mark a tremendous milestone in gene therapy for hereditary hearing loss.

In summary, we report the tissue tropism patterns of three AAV serotypes, DJ, DJ8, and PHP.B, in the murine inner ear after inoculation through the RWM directly into the scala tympani. Our findings will aid additional studies focusing on hearing loss genes that play essential roles in K^+ recycling by degeneration and/or abnormality of non-sensory cells and the stria vascularis of the cochlea. Further preclinical studies using large animals and non-human primates are required to fully evaluate the effectiveness of AAV infection before its application in human clinical trials, although our findings may enable the development of better treatment strategies for improving the quality of life of patients with genetic hearing loss.

MATERIALS AND METHODS

rAAV

The rAAV constructs used in the present study, containing the inverted terminal repeat of AAV serotype 2 and the capsid of AAV serotypes DJ, DJ8, and PHP.B, with a CMV-driven *EGFP* transgene cassette, were obtained from SignaGen Laboratories (Rockville, MD, USA). Titers of stocks were maintained at 1.0×10^{13} viral genomes [VGs]/mL for each virus and were stored at $-80°C$ until use.

Inoculation of rAAV

The Institute for Cancer Research (ICR) mice were purchased from Hyochang Science (Daegu, Republic of Korea), and they were maintained free of any known and suspected murine pathogens in a pathogen-free animal facility. All animal protocols were approved by the Institutional Animal Care and Use Committee of Kyungpook National University.

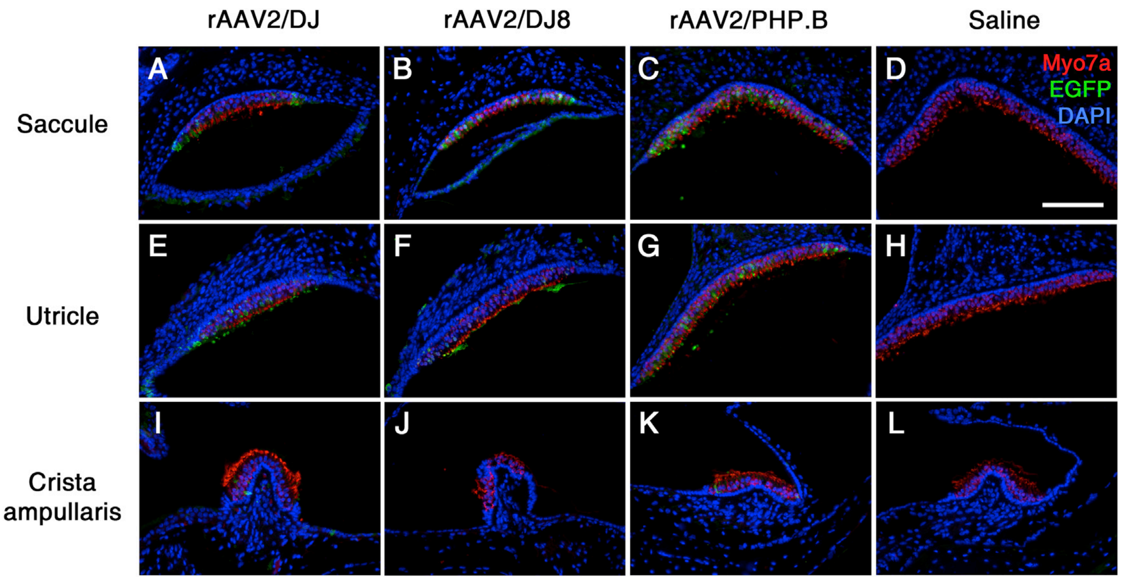

Figure 5. rAAV Transduction into the Vestibule

EGFP immunoreactivity (green) in cross-sections of the saccule (A–D), utricle (E–H), and crista ampullaris (I–L) in mice at 3 weeks of age after infection with the indicated vectors. Vestibular hair cells and nuclei were stained with Myo7a (red) and DAPI (blue), respectively. Representative images of six, four, and three replicates, respectively, are shown. Scale bar represents 100 μm.

Surgeries for rAAV inoculation were performed on the left ears of the mice; the right ears served as non-surgery controls. Pups at postnatal day 2 were anesthetized using hypothermia and transferred to clean ice. Before each operation, 70% ethanol was used to gently wipe the postauricular area. All procedures were performed in a dedicated workspace using sterile techniques and a surgical microscope. A 5-mm incision was made approximately 2 mm away from the auricular crease, and muscles were bluntly separated to expose the RWM. A small hole in the RWM was generated, through which 1 μL (1.0×10^{10} VG/mL) rAAV2/DJ, rAAV2/DJ8, or rAAV2/PHP.B was injected. After inoculation, the exposed RWM was irrigated with prewarmed normal saline, and the incision was closed with surgical sutures. Each surgical procedure lasted approximately 20 min.

ABR

At 3 weeks post-infection, hearing thresholds of the mice were determined in a sound-proofed room based on ABR recordings (System 3 ABR Workstation; Tucker-Davis Technologies, Alachua, FL, USA), as previously described.[41] Mice were anesthetized with a mixture of alfaxan (4 mg/100 g) and xylazine hydrochloride (0.13 mg/100 g) by intramuscular injection and placed on a heating pad. Subcutaneous needle electrodes were inserted into the vertex (channel), ipsilateral ear (reference), and contralateral ear (ground). Acoustic stimuli were applied monaurally through a speaker, and recordings were made in response to click and tone burst stimuli at frequencies of 8, 16, and 32 kHz. Stimuli were delivered at an amplitude of 90-dB sound pressure level, and amplitudes were reduced in 5-dB decrements to determine acoustic thresholds.

Histology

Fixation

Mice were anesthetized as described above and fixed by cardiac perfusion with 4% paraformaldehyde (PFA; pH 7.4) in PBS. Inner ears were isolated from temporal bones and fixed by submersion in 4% PFA in PBS.

Paraffin Sections

Fixed inner ears were decalcified with 10% EDTA in PBS for 2 days at 4°C, dehydrated in a graded ethanol series, permeabilized with xylene, and embedded in paraffin at room temperature (RT). The paraffin-embedded inner ears were then serially sectioned into 5-μm slices using a microtome (Leica RM2235; Leica Microsystems, Bensheim, Germany). All tissue sections were mounted on Superfrost Plus microscope slides (Thermo Fisher Scientific, Pittsburgh, PA, USA). Slides with paraffin sections were stored at RT until use.

Cochlear Whole Mounts

The organ of Corti was prepared from the inner ears of the ICR mice. The isolated inner ears were quickly fixed by injecting 4% PFA in PBS through the oval window and immersing them in the same fixative for 2 h at 4°C. After Reissner's membrane and the lateral wall and tectorial membrane of the cochlea were removed, the organ of Corti was dissected into individual turns.

Immunofluorescence

Immunofluorescence assays were conducted as described previously,[9] with minor modifications: primary rabbit anti-Myo7a (Abcore,

Table 1. Transduction Patterns of AAV Serotypes DJ, DJ8, and PHP.B in the Inner Ear Cells of the Cochlea

Serotype	Cochlear Cell Type	Cochlear Cell Type with Highest Transduction Rate
rAAV2/DJ	inner hair cells	inner hair cells
	outer hair cells	outer hair cells
	supporting cells	supporting cells
	Reissner's membrane	Reissner's membrane
	interdental cells	interdental cells
	root cells	root cells
rAAV2/DJ8	inner hair cells	inner hair cells
	outer hair cells	outer hair cells
	supporting cells	
	Reissner's membrane	
	root cells	
rAAV2/PHP.B	inner hair cells	inner hair cells
	outer hair cells	outer hair cells
	supporting cells	
	Reissner's membrane	

Ramona, CA, USA), rabbit anti-GFP (Life Technologies, La Jolla, CA, USA), and mouse anti-GFP antibody (Millipore, Billerica, MA, USA) were diluted to 1:500, 1:500, and 1:200 in blocking solution, respectively. Secondary Alexa Fluor 555-conjugated goat anti-rabbit immunoglobulin G (IgG), Alexa Fluor 488-conjugated goat anti-rabbit IgG, and Alexa Fluor 488-conjugated goat anti-mouse IgG antibody (Invitrogen, La Jolla, CA, USA) were diluted to 1:1,000 in blocking solution, respectively. Nuclei were stained with 1 μg/mL DAPI solution diluted in methanol for 5 min at RT. The samples were washed with PBS to remove any residual antibodies and mounted with Fluoromount (Sigma-Aldrich, St. Louis, MO, USA). Images were captured using a fluorescence microscope (Axio Imager A2; Carl Zeiss, Oberkochen, Germany).

Cell Counts and Transduction Efficiency Analysis

Hair cells expressing EGFP were counted per 200-μm portions of each cochlear turn: the apex was defined as the top 15%, the mid as the mid 50%, and the base as the lower 85% of the distance from the apical tip of the cochlea. Counts were converted to a percentage of total hair cells. Any segments with dissection-related damage were omitted from the analysis.

Statistics

ABR and cell-counting data were presented as mean values ± SD and mean values, respectively. Statistical analysis was performed using the Prism 5 software package (GraphPad, San Diego, CA, USA). Two groups were compared using unpaired two-tailed Student's t test. For comparisons of more than two groups, one-way ANOVA was performed and followed by a post hoc test with Bonferroni correction of pairwise group differences. p values of < 0.05 were considered significant.

SUPPLEMENTAL INFORMATION

Supplemental Information includes three figures and can be found with this article online at https://doi.org/10.1016/j.omtm.2019.01.002.

AUTHOR CONTRIBUTIONS

U.-K.K. and J.B. designed and conceptualized experiments. M.-A.K., N.R., H.-M.K., Y.-R.K., B.L., and T.-J.K. performed experiments and data analysis. M.-A.K., N.R., and U.-K.K. wrote the manuscript.

CONFLICTS OF INTEREST

The authors have declared that no conflicts of interest exist.

ACKNOWLEDGMENTS

Our research was supported by National Research Foundation of Korea, Republic of Korea: Grant 2018R1A2B2004606 (to U.-K.K.), 2014M3A9D5A01073865 (to U.-K.K. and J.B.), and 2017R1A2B3009133 (to J.B.). The Korea Health Technology R&D Project through the Korea Health Industry Development Institute (KHIDI) funded by the Ministry of Health & Welfare, Republic of Korea, also supported this study: HI18C0160 (to U.-K.K).

REFERENCES

1. Smith, R.J., Bale, J.F., Jr., and White, K.R. (2005). Sensorineural hearing loss in children. Lancet *365*, 879–890.
2. Forge, A., and Wright, T. (2002). The molecular architecture of the inner ear. Br. Med. Bull. *63*, 5–24.
3. Zhang, W., Kim, S.M., Wang, W., Cai, C., Feng, Y., Kong, W., and Lin, X. (2018). Cochlear Gene Therapy for Sensorineural Hearing Loss: Current Status and Major Remaining Hurdles for Translational Success. Front. Mol. Neurosci. *11*, 221.
4. Akil, O., Seal, R.P., Burke, K., Wang, C., Alemi, A., During, M., Edwards, R.H., and Lustig, L.R. (2012). Restoration of hearing in the VGLUT3 knockout mouse using virally mediated gene therapy. Neuron *75*, 283–293.
5. Askew, C., Rochat, C., Pan, B., Asai, Y., Ahmed, H., Child, E., Schneider, B.L., Aebischer, P., and Holt, J.R. (2015). Tmc gene therapy restores auditory function in deaf mice. Sci. Transl. Med. *7*, 295ra108.
6. Chang, Q., Wang, J., Li, Q., Kim, Y., Zhou, B., Wang, Y., Li, H., and Lin, X. (2015). Virally mediated Kcnq1 gene replacement therapy in the immature scala media restores hearing in a mouse model of human Jervell and Lange-Nielsen deafness syndrome. EMBO Mol. Med. *7*, 1077–1086.
7. Chien, W.W., Isgrig, K., Roy, S., Belyantseva, I.A., Drummond, M.C., May, L.A., Fitzgerald, T.S., Friedman, T.B., and Cunningham, L.L. (2016). Gene Therapy Restores Hair Cell Stereocilia Morphology in Inner Ears of Deaf Whirler Mice. Mol. Ther. *24*, 17–25.
8. Yu, Q., Wang, Y., Chang, Q., Wang, J., Gong, S., Li, H., and Lin, X. (2014). Virally expressed connexin26 restores gap junction function in the cochlea of conditional Gjb2 knockout mice. Gene Ther. *21*, 71–80.
9. Kim, M.A., Cho, H.J., Bae, S.H., Lee, B., Oh, S.K., Kwon, T.J., Ryoo, Z.Y., Kim, H.Y., Cho, J.H., Kim, U.K., and Lee, K.Y. (2016). Methionine Sulfoxide Reductase B3-Targeted In Utero Gene Therapy Rescues Hearing Function in a Mouse Model of Congenital Sensorineural Hearing Loss. Antioxid. Redox Signal. *24*, 590–602.
10. Dalkara, D., Goureau, O., Marazova, K., and Sahel, J.A. (2016). Let There Be Light: Gene and Cell Therapy for Blindness. Hum. Gene Ther. *27*, 134–147.
11. Wu, Z., Asokan, A., and Samulski, R.J. (2006). Adeno-associated virus serotypes: vector toolkit for human gene therapy. Mol. Ther. *14*, 316–327.
12. Grimm, D., Pandey, K., Nakai, H., Storm, T.A., and Kay, M.A. (2006). Liver transduction with recombinant adeno-associated virus is primarily restricted by capsid serotype not vector genotype. J. Virol. *80*, 426–439.

13. Grimm, D., and Kay, M.A. (2003). From virus evolution to vector revolution: use of naturally occurring serotypes of adeno-associated virus (AAV) as novel vectors for human gene therapy. Curr. Gene Ther. *3*, 281–304.

14. Hoggan, M.D., Blacklow, N.R., and Rowe, W.P. (1966). Studies of small DNA viruses found in various adenovirus preparations: physical, biological, and immunological characteristics. Proc. Natl. Acad. Sci. USA *55*, 1467–1474.

15. Parks, W.P., Melnick, J.L., Rongey, R., and Mayor, H.D. (1967). Physical assay and growth cycle studies of a defective adeno-satellite virus. J. Virol. *1*, 171–180.

16. Bantel-Schaal, U., and zur Hausen, H. (1984). Characterization of the DNA of a defective human parvovirus isolated from a genital site. Virology *134*, 52–63.

17. Atchison, R.W., Casto, B.C., and Hammon, W.M. (1965). Adenovirus-Associated Defective Virus Particles. Science *149*, 754–756.

18. Gao, G., Vandenberghe, L.H., Alvira, M.R., Lu, Y., Calcedo, R., Zhou, X., and Wilson, J.M. (2004). Clades of Adeno-associated viruses are widely disseminated in human tissues. J. Virol. *78*, 6381–6388.

19. Gao, G.P., Alvira, M.R., Wang, L., Calcedo, R., Johnston, J., and Wilson, J.M. (2002). Novel adeno-associated viruses from rhesus monkeys as vectors for human gene therapy. Proc. Natl. Acad. Sci. USA *99*, 11854–11859.

20. Koerber, J.T., Jang, J.H., and Schaffer, D.V. (2008). DNA shuffling of adeno-associated virus yields functionally diverse viral progeny. Mol. Ther. *16*, 1703–1709.

21. Maheshri, N., Koerber, J.T., Kaspar, B.K., and Schaffer, D.V. (2006). Directed evolution of adeno-associated virus yields enhanced gene delivery vectors. Nat. Biotechnol. *24*, 198–204.

22. Grimm, D., Lee, J.S., Wang, L., Desai, T., Akache, B., Storm, T.A., and Kay, M.A. (2008). In vitro and in vivo gene therapy vector evolution via multispecies interbreeding and retargeting of adeno-associated viruses. J. Virol. *82*, 5887–5911.

23. Deverman, B.E., Pravdo, P.L., Simpson, B.P., Kumar, S.R., Chan, K.Y., Banerjee, A., Wu, W.L., Yang, B., Huber, N., Pasca, S.P., and Gradinaru, V. (2016). Cre-dependent selection yields AAV variants for widespread gene transfer to the adult brain. Nat. Biotechnol. *34*, 204–209.

24. Sayyid, Z.N., Kim, G.S., and Cheng, A.G. (2018). Molecular therapy for genetic and degenerative vestibular disorders. Curr. Opin. Otolaryngol. Head Neck Surg. *26*, 307–311.

25. Kilpatrick, L.A., Li, Q., Yang, J., Goddard, J.C., Fekete, D.M., and Lang, H. (2011). Adeno-associated virus-mediated gene delivery into the scala media of the normal and deafened adult mouse ear. Gene Ther. *18*, 569–578.

26. Praetorius, M., Brough, D.E., Hsu, C., Plinkert, P.K., Pfannenstiel, S.C., and Staecker, H. (2009). Adenoviral vectors for improved gene delivery to the inner ear. Hear. Res. *248*, 31–38.

27. Shibata, S.B., Yoshimura, H., Ranum, P.T., Goodwin, A.T., and Smith, R.J.H. (2017). Intravenous rAAV2/9 injection for murine cochlear gene delivery. Sci. Rep. *7*, 9609.

28. Suzuki, J., Hashimoto, K., Xiao, R., Vandenberghe, L.H., and Liberman, M.C. (2017). Cochlear gene therapy with ancestral AAV in adult mice: complete transduction of inner hair cells without cochlear dysfunction. Sci. Rep. *7*, 45524.

29. György, B., Sage, C., Indzhykulian, A.A., Scheffer, D.I., Brisson, A.R., Tan, S., Wu, X., Volak, A., Mu, D., Tamvakologos, P.I., et al. (2017). Rescue of Hearing by Gene Delivery to Inner-Ear Hair Cells Using Exosome-Associated AAV. Mol. Ther. *25*, 379–391.

30. Pan, B., Askew, C., Galvin, A., Heman-Ackah, S., Asai, Y., Indzhykulian, A.A., Jodelka, F.M., Hastings, M.L., Lentz, J.J., Vandenberghe, L.H., et al. (2017). Gene therapy restores auditory and vestibular function in a mouse model of Usher syndrome type 1c. Nat. Biotechnol. *35*, 264–272.

31. Iizuka, T., Kamiya, K., Gotoh, S., Sugitani, Y., Suzuki, M., Noda, T., Minowa, O., and Ikeda, K. (2015). Perinatal Gjb2 gene transfer rescues hearing in a mouse model of hereditary deafness. Hum. Mol. Genet. *24*, 3651–3661.

32. Isgrig, K., Shteamer, J.W., Belyantseva, I.A., Drummond, M.C., Fitzgerald, T.S., Vijayakumar, S., Jones, S.M., Griffith, A.J., Friedman, T.B., Cunningham, L.L., and Chien, W.W. (2017). Gene Therapy Restores Balance and Auditory Functions in a Mouse Model of Usher Syndrome. Mol. Ther. *25*, 780–791.

33. Yeh, T.H., and Tran Ba Huy, P. (1992). Contribution of cell culture to the study of the physiology of the stria vascularis and Reissner's membrane. Acta Otolaryngol. *112*, 254–258.

34. Mira, E. (1971). The function of the interdental cells of the limbus spiralis. A cytoenzymological study. Pract. Otorhinolaryngol. (Basel) *33*, 252–259.

35. Jagger, D.J., and Forge, A. (2013). The enigmatic root cell - emerging roles contributing to fluid homeostasis within the cochlear outer sulcus. Hear. Res. *303*, 1–11.

36. Hilgert, N., Smith, R.J., and Van Camp, G. (2009). Function and expression pattern of nonsyndromic deafness genes. Curr. Mol. Med. *9*, 546–564.

37. Kern, A., Schmidt, K., Leder, C., Müller, O.J., Wobus, C.E., Bettinger, K., Von der Lieth, C.W., King, J.A., and Kleinschmidt, J.A. (2003). Identification of a heparin-binding motif on adeno-associated virus type 2 capsids. J. Virol. *77*, 11072–11081.

38. Landegger, L.D., Pan, B., Askew, C., Wassmer, S.J., Gluck, S.D., Galvin, A., Taylor, R., Forge, A., Stankovic, K.M., Holt, J.R., and Vandenberghe, L.H. (2017). A synthetic AAV vector enables safe and efficient gene transfer to the mammalian inner ear. Nat. Biotechnol. *35*, 280–284.

39. Hordeaux, J., Wang, Q., Katz, N., Buza, E.L., Bell, P., and Wilson, J.M. (2018). The Neurotropic Properties of AAV-PHP.B Are Limited to C57BL/6J Mice. Mol. Ther. *26*, 664–668.

40. György, B., Meijer, E.J., Ivanchenko, M.V., Tenneson, K., Emond, F., Hanlon, K.S., Indzhykulian, A.A., Volak, A., Karavitaki, K.D., Tamvakologos, P.I., et al. (2018). Gene Transfer with AAV9-PHP.B Rescues Hearing in a Mouse Model of Usher Syndrome 3A and Transduces Hair Cells in a Non-human Primate. Mol. Ther. Methods Clin. Dev. *13*, 1–13.

41. Lee, H.J., Yoo, S.J., Lee, S., Song, H.J., Huh, M.I., Jin, S.U., Lee, K.Y., Lee, J., Cho, J.H., and Chang, Y. (2012). Functional activity mapping of rat auditory pathway after intratympanic manganese administration. Neuroimage *60*, 1046–1054.

OMTM, Volume *13*

Supplemental Information

Targeted Gene Delivery into the Mammalian Inner Ear Using Synthetic Serotypes of Adeno-Associated Virus Vectors

Min-A Kim, Nari Ryu, Hye-Min Kim, Ye-Ri Kim, Byeonghyeon Lee, Tae-Jun Kwon, Jinwoong Bok, and Un-Kyung Kim

Supplementary Figures

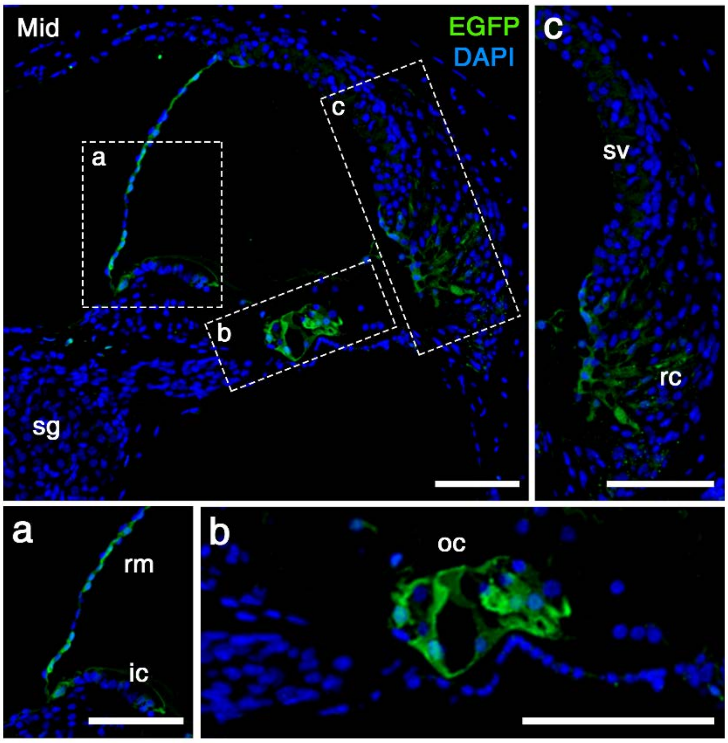

Supplementary Figure 1. Transduction of rAAV2/DJ into murine cochlea. EGFP immunoreactivity (green) in cross-sections of cochlear middle turns in 3-week-old mice infected with rAAV2/DJ. High-magnification images of Reissner’s membrane, interdental cells, organ of Corti, root cells, and stria vascularis are shown (a–c, respectively). Cell nuclei were stained with DAPI (blue). ic, interdental cell; oc, organ of Corti; rc, root cell; rm, Reissner’s membrane; sg, spiral ganglion; sv, stria vascularis. Scale bars represent 100 µm.

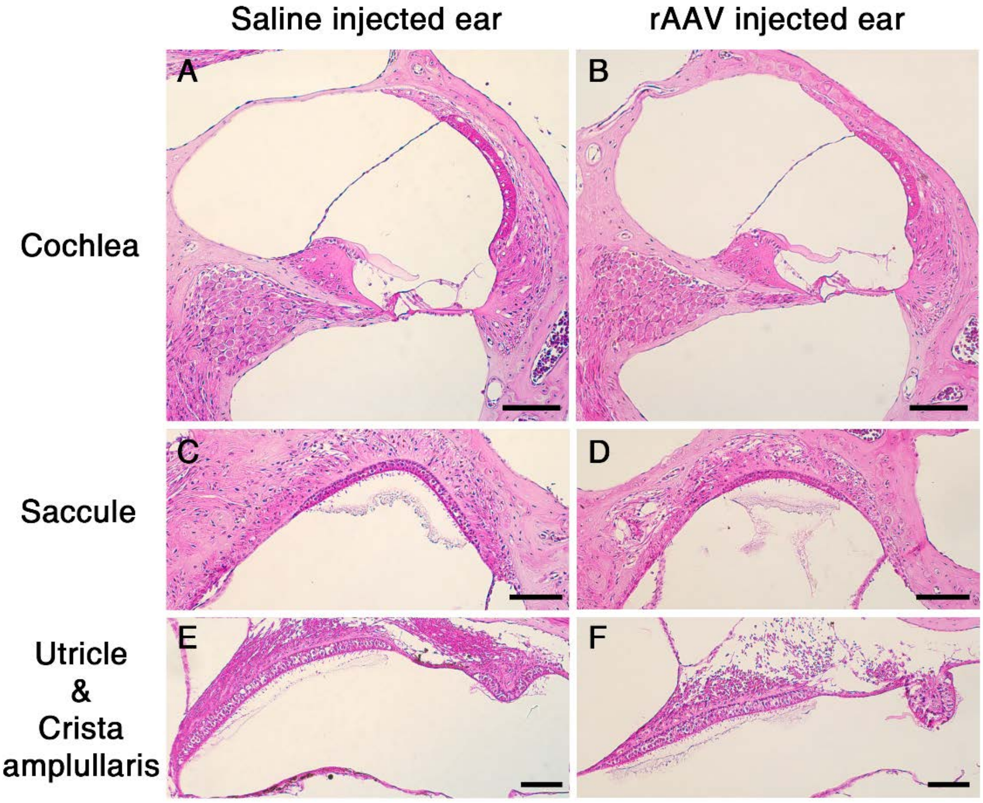

Supplementary Figure 2. Histological observations of the utricle, saccule, and crista ampullaris. Cross-sections of the (A and B) cochlear middle turn, (C and D) saccule, (E and F) utricle and crista ampullaris from saline and rAAV injected ears of mice were obtained at 3 weeks of age and stained with hematoxylin-eosin. Representative images of three replicates each are shown. Scale bars: 100 µm.

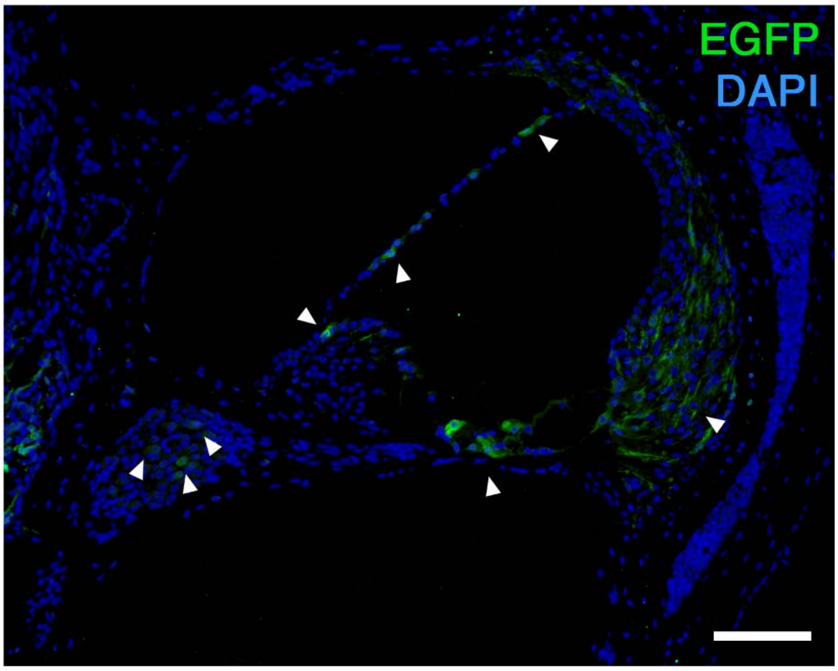

Supplementary Figure 3. EGFP expression in the cochlea infected by rAAV2/PHP.B. EGFP immunoreactivity (green) in cross-sections of cochlear basal turns in mice 3 weeks of age are shown. Nuclei were stained with DAPI (blue). White arrowheads indicate representative EGFP-expressing cells. Scale bar represents 100 μm.
